# Voluntary Additional Welfare Monitoring of Farm Animals Used in Research: Maximising Benefits Requires Sustained Support

**DOI:** 10.3390/ani15192817

**Published:** 2025-09-26

**Authors:** Siobhan Mullan, Jessica Stokes, Helena Elizabeth Hale, Timm Konold

**Affiliations:** 1 School of Veterinary Medicine, University College Dublin, D04 V1W8 Dublin, Ireland; 2 Agricultural Sciences and Practice, Royal Agricultural University, Cirencester GL7 6JS, UK; jessica.stokes@rau.ac.uk; 3 Bristol Vet School, University of Bristol, Avon BS40 5DU, UK; helena.hale@bristol.ac.uk; 4 Animal and Plant Health Agency, Surrey KT153NB, UK; timm.konold@apha.gov.uk

**Keywords:** animal welfare, experimental animals, welfare assessment, co-creation, innovation

## Abstract

**Simple Summary:**

Monitoring animal welfare is a key element of conducting research involving animals. The aim of this project was to co-create animal welfare monitoring systems that could contribute to best practice husbandry standards of farm animals in a real animal research setting. Researchers worked with nine staff to co-design six bespoke welfare assessment protocols to be conducted in addition to legally required welfare monitoring for adult cattle, calves, sheep, pigs, and goats in specific experimental environments that included both positive and negative welfare elements. Four protocols were subsequently applied with variable frequency by three staff to cattle, goats, and two pig populations. Assessments were all observational and included behavioural and physical condition data. Two staff provided feedback on their views of the process. A key finding was that with facilitation, staff could generate protocols that included elements designed to encourage or evaluate interventions to promote positive emotions. However, data collection was sporadic, and although the staff who provided feedback reported that they valued the process highly, they noted that the primary challenge was finding the time to conduct the additional assessments. We therefore conclude that sustained support is likely to be required to maximise the benefits for the animals and staff of developing and conducting additional voluntary welfare monitoring of farm animals.

**Abstract:**

The aim of this project was to co-create an animal welfare monitoring system that incorporated both positive and negative welfare measures that would contribute to best practice husbandry standards of farm animals in a real animal research setting. Researchers worked with nine staff to co-design six bespoke welfare assessment protocols to be conducted in addition to legally required welfare monitoring for adult cattle, calves, sheep, pigs, and goats in specific experimental environments. Four protocols were subsequently applied with variable frequency by three staff to cattle, goats, and two pig populations. Assessments were all observational, and included behavioural scan sampling, Qualitative Behaviour Assessment scores, visual analogue mood scores, and physical condition data. Two staff provided feedback on their views of the process. A key finding was that with facilitation, staff could generate protocols that included elements designed to encourage or evaluate interventions to promote positive emotions. However, data collection was sporadic, and although the staff who provided feedback reported that they valued the process highly, they noted that the primary challenge was finding the time to conduct the assessments. We therefore conclude that sustained support is likely to be required to maximise the benefits for the animals and staff of developing and conducting voluntary welfare monitoring of farm animals.

## 1. Introduction

Guiding principles and welfare frameworks such as the 3Rs [1] exist for the protection of animals used for research purposes, requiring that animal suffering and animal use are minimised, and that animal health and wellbeing are promoted. An ever-growing body of research investigates and promotes improved welfare assessment of animals used in laboratory settings [2]. However, there is a gap in the study of farm animals kept for research, and, in particular, appropriate methods of assessing and promoting positive experiences, which needs to be filled to better understand and advance their welfare [3]. Much of the previous focus has been on health management of the animals; for example, a Federation of European Laboratory Animal Science Associations (FELASA) Working Group has provided recommendations for the health monitoring of ruminants used in research [4], or for care relating to specific procedures, for example, pig undergoing laryngeal transplantation [5]. One study reported using focus groups to enhance pig welfare in a large research organisation through developing 12 recommendations relating to behavioural management, housing, research procedures, transportation, and rehoming programmes. The authors proposed methods for implementation and monitoring across the organisation in the future [6].

Failing to address the challenges that can impact animal welfare may concern ethics, the law, reputation and public perception, as well as the quality, validity, and reliability of outputs from animal research [7]. When it comes to public perception, farm animal welfare is an area of concern, with, for example, 91% of European Union (EU) citizens believing it important to protect farm animal welfare [8]. Furthermore, public opinion polls on the use of animals in scientific research reveal that 70% of adults in EU member states agree that ‘enabling the full replacement of all forms of animal testing with non-animal testing methods should be a priority for the EU’ [9]. Staff who provide animal husbandry have daily contact with the animals under their care and are therefore well-positioned to influence overall welfare. However, they may lack the resources (such as enrichments, space or outdoor access to provide for animals) and institutional support (such as time to conduct assessments) needed to provide best practice in animal welfare.

This study was carried out in a UK research establishment that keeps several farm animal species for a variety of scientific research purposes. They had identified an internal need for additional welfare monitoring protocols to enhance existing practices and instigated a collaborative project. This provided a unique opportunity to co-design an innovative illustrative example of welfare assessments for farm animals species in different experimental settings. An innovation can be defined as ‘a new idea that proves successful in practice’ [10]. In contrast to ‘linear’ innovation (standard knowledge transfer methods), ‘interactive’ innovation, jointly created between practitioners and researchers and other actors as appropriate, ‘tend to deliver solutions that are well adapted to circumstances and which are easier to implement since the participatory process is favourable to speeding up the introduction, dissemination, and acceptance of the new ideas’ [10]. Co-creation of welfare assessments and voluntary monitoring has been shown to be effective in improving the welfare of working equids [11], and practice-led co-innovation is advocated for farm animal welfare initiatives [12]. However, ‘interactive’ innovation initiatives have not been reported for research animals, although some training interventions in adopting new practices, such as rat tickling, have been evaluated [13]. This study aimed to facilitate staff at an organisation level to co-develop and trial welfare assessment protocols that were applicable in addition to statutory daily husbandry and welfare checks, with a particular focus on positive experiences and enrichment. It was essential that the protocols were both practical and therefore feasible to conduct alongside existing animal care duties. Permanent housing of large animals used for research, which it also applies to commercial farming of pigs and cattle, may cause welfare challenges because of space restrictions and the lack of environmental stimuli associated with living outdoors. Welfare assessment protocols may provide evidence for the existence of welfare issues so that actions can be taken to improve animal welfare.

## 2. Materials and Methods

All studies involving animals were approved by the UK Home Office in the appropriate project licences following internal review by the animal welfare ethical review body of the research establishment, which included welfare assessments as stipulated in the respective project licence. The specific welfare assessment trials described here are in addition and were not subject to ethical review. Informed consent was received by participants to publish their feedback.

### Welfare Assessment Protocol Development

The researchers (from the field of farmed animal welfare science) worked with staff at the research establishment to co-create additional welfare assessment protocols which would address the needs of each unit and would be feasible to conduct. The staff were all animal husbandry staff with experience in the relevant species who were licenced to carry out regulated procedures under Animals (Scientific Procedures) Act 1986 in the relevant species and/or Named Animal Care and Welfare Officers. An initial scoping visit was conducted in May 2018, followed by a subsequent workshop for nine members of staff in June 2018 to support the creation of unit-specific protocols for different species. Here, small groups of staff (three per group) from species-specific work units were facilitated in separate workshops through four exercises, which were

1. What is good animal welfare?;
2. What would be the benefits of monitoring welfare?;
3. Identify focus of assessments—Concerns over welfare, areas to monitor;
4. What/Who/When/How for each measure.

Results of the workshop were then used to create the additional voluntary welfare assessment protocols for pigs (two sites), adult cattle and goats in a long-term enclosed housing unit, 6-month-old calves at pasture, and sheep housed in an open barn. The protocols for each species are available in Supplementary Information. The protocols were not harmonised between species, as the aim was to maximise the sense of ownership each group felt over their protocol to try to increase the likelihood of implementation within each unit. Each additional voluntary welfare monitoring protocol was designed to be feasible to undertake as part of the working routine, aiming to take less than 20 min each, and the staff had control over when assessments would be made to fit in with their working day, again to promote implementation. Staff completed a training and standardisation session in the protocol relevant to their role, lasting approximately 1 h, but formal inter-observer reliability testing was not conducted. The protocols were subsequently applied by staff during a 13-month period. Reflections on staff experience were collected 17 months later via a short online survey. The length of time before collecting reflections was due to continued organisational discussions about when to conclude the initiative. Feedback consisted of responses to the following questions:

1. Thinking about the process as a whole, please rate the following statements (5-point scale from completely disagree to completely agree):
  - The protocol reflected my ideas in the workshop;
  - The assessments were easy to carry out;
  - I had enough time to conduct the assessments;
  - Doing the assessments positively influenced how I worked with the animals;
  - Reviewing the data is useful in understanding the welfare of the animals;
  - I would recommend developing and conducting similar welfare assessments to a colleague in a similar position.
2. Imagine a colleague of yours at a similar institute asks whether they should get involved in an equivalent initiative. What would you say would be the positives about doing so?
3. And what would you tell your colleague might be some of the challenges?
4. Please provide any comments on how any part of the process could be improved.
5. Do you have any other comments?

## 3. Results

### 3.1. Scoping Exercise

Within the scoping exercise, different animal units were visited by the researchers and initial ideas about welfare assessment priorities were suggested based on existing husbandry practice. Project aspirations were identified with the main aim being to create a welfare assessment that could be used to (1) engage staff with the welfare assessment process, (2) identify specific welfare concerns or opportunities to improve welfare for individual animals/groups, and (3) test the interest and preferences of enrichment resources provided to the animals, which would enable farm staff to adapt and adjust enrichments accordingly. A capacity of one 15 min observation per week per unit was outlined for possible application of the additional voluntary welfare assessment protocols. These assessments would be conducted in addition to their twice daily welfare monitoring that they already had a duty to conduct.

### 3.2. Workshop Outcomes

The half-day consultation workshops started with discussions of what constitutes animal welfare in general, where participants considered good physical condition and appropriate behaviour to be important, including elements relating to natural behaviours such as wallowing for pigs. Absence of negative states, such as lameness or respiratory disease, as well as positive welfare, such as enjoying interacting with enrichments, were raised. Many benefits of monitoring welfare were raised, including upskilling staff in understanding unfamiliar species through behavioural observations (e.g., goats), evaluating animal preferences for different food and physical enrichments, monitoring the impact of procedural interventions such as blood sampling, and identifying when animals reach thresholds for interventions to improve welfare. In addition, it was recognised that longitudinal monitoring of welfare could provide information about the welfare of animals over time.

Staff identified which animal units would be involved in the welfare assessment project. A particular knowledge gap was identified for goats, so they were chosen alongside pigs, sheep, and cattle to develop understanding of their behaviour. Discussions culminated in the creation of additional voluntary welfare assessment protocols by researchers SM and JS (see Supplementary Information) for each participating unit to take forward. Staff identified pig behaviour (in general and also in response to handling), lameness, and injuries as key areas for assessment. In addition, it was perceived that some of the sows were becoming fat, impeding the ease of taking blood samples and potentially reducing welfare; therefore, body condition and skin thickness were also included. The focus of the goats and adult sheep was on their behavioural repertoire and response to enrichment and overall quality of life assessment as they were housed long-term and potential disease models. Groups of calves were regularly brought to the site, and the staff were keen to monitor the impact of collection, mixing, and transport, including through formal health recording. Finally, the general behaviour and response to enrichment of the sheep was of particular interest. Where possible, existing measures (e.g., AssureWel assessments for injury and lameness in pigs [14]) were integrated into the overall protocol.

### 3.3. Protocols and Assessments

The additional voluntary welfare assessments were conducted sporadically and variably for each unit. The existing mandatory daily welfare monitoring continued as usual. Only three of the nine staff conducted any assessments at all, despite the expectation at the outset that everyone would be taking part in observations and apparent enthusiasm for doing so during the workshop.

### 3.4. Pigs

#### 3.4.1. Animal Populations and Husbandry

Two study populations were assessed in different housing units (Unit A, *n* = 4, Large White × Landrace sows; Unit B, *n* = 15, Large White sows). The four sows at the Unit A (used for occasional blood harvesting) were kept together in a large indoor pen with deep bedded straw and enrichment objects that included wood and branches. They had access to an outdoor grassed paddock every day for several hours, with a mud wallow and rooting area, a tree for shade, branches, and feeding enrichment balls which could be filled with hay or food treats. The fifteen pigs in the Unit B breeding unit were inbred to generate a uniform major histocompatibility complex region [15]. They were kept in small groups indoors in large deep bedded straw pens but had access to an outdoor area with a grassy bank and mud wallow, which was provided daily in rotation so that each group had access to the outdoor area once a week. All pigs in both units were fed a commercial concentrate diet twice a day.

#### 3.4.2. Data Collection

In each unit, data collection was conducted by a staff member responsible for the animals and consisted of behavioural scan sample observations (all pigs observed every 15 s, for a total of 10 min); physical condition data (lameness; skin condition; body marks); neck skin thickness (as an indicator of subcutaneous fat and how difficult, and therefore aversive, it might be to perform a blood sample); weight tape; body condition score; handling stress); a single qualitative mood score on a scale of 1–20; response to blood sampling training score (the training involved clicker training with food rewards to get the pig into the required position for sampling); opportunities for pleasure/suggestions for change between assessments. See further descriptions in the Supplementary Material (Pigs—Welfare Assessment Protocols).

#### 3.4.3. Behavioural Observations

In total, 70 scan sample observations sessions were made of the pig population at Unit B and 23 were made at Unit A; results for both study populations are summarised in Figure S1 (Supplementary Material). In comparison to the sows at Unit A, the pigs at Unit B were less often observed engaged in ‘exploratory behaviour towards manipulable material’ (10% vs. 64% of scans) and ‘eating’ (6% vs. 16% of scans), and were more often observed ‘resting’ (53% vs. 15% of scans) and ‘exploring pen fixtures and fittings’ (30% vs. 1% of scans).

#### 3.4.4. Physical Condition

The most comprehensive physical condition data were collected at Unit B (15 animals were observed between four and seven occasions, total *n* = 70 observations over a period of 5 months) where the main physical problems identified were mild (Score 1) gait abnormalities (56% of observations) and mild (Score 1) skin lesions (51% of observations). Worse scores of 2 or more for any condition were never observed in these animals. In addition, handling stress was always scored 0/4, response to training was always scored 18/20, and the mean qualitative mood score (scale of 1—worst mood to 20—best mood) was 18.0, with a range of 18–19. These pigs were never assessed using the weight tape or for skin thickness. The four pigs at Unit A were only scored for physical condition on three occasions during the first month. During these observations all four sows were recorded as score 1 for lameness, and two sows had a small skin mark on one occasion. Neck skin thickness was recorded on a single occasion as 9, 9, 11, and 6 mm. The weight tape was not used, and training was not undertaken; therefore, the response was not scored. Qualitative mood scores (1–20 scale) were conducted on the four animals on 20 days over a 6-month period with a mean score of 18.7, range 10–20.

### 3.5. Cattle

#### 3.5.1. Animal Population and Husbandry

Sixteen castrated male animals (seven Holstein Friesians or crossbreds between 77 and 91 months of age and nine 5–6-month-old Hereford crossbreds) used for a study to investigate transmissibility of various transmissible spongiform encephalopathy agents were assessed, housed in five enclosed pens within a large barn in Unit C. Two pens had animals housed in pairs, and the remaining pens had groups of three, four, and five animals. Pens were 7.1 m wide × 7.8 m long, and enrichment provided included a wall brush, gate brush (both were dual brush systems where one brush was attached vertically to the pen wall, whereas the other brush was spring mounted horizontally at cattle height so that animals could scratch sides and backs), rubber balls that made a squeak when pressed, and straw clumps that could be manipulated/spread around. Hay was provided ad libitum, a commercial concentrate ration was fed twice daily, and they were kept on straw bedding.

#### 3.5.2. Data Collection

Data collection was conducted by one staff member and consisted of scan sample observations to provide behavioural time budgets against an ethogram (cows observed in turn every 15 s, for a total of 10 min); Qualitative Behavioural Assessment (QBA) score for 20 terms [16] on 120 mm visual analogue scales; overall mood score recorded on 120 mm visual analogue scale; willingness of assessor to take on the life of the animal (20-point scale: 1 = extremely unwilling, 20 = extremely willing); and opportunities for pleasure/suggestions for change between assessments (these suggestions were at the observer’s discretion, formally recording any ideas they had). See further descriptions in the Supplementary Material (Cattle—Welfare Assessment Protocols).

#### 3.5.3. Behavioural Observations

In total, behaviour of the 16 adult cattle was assessed by scan sampling on 27 occasions in unit C. On two occasions, some individual animals or pens were not able to be observed giving a total number of animal observation sessions of 420. The results of the behavioural observations are shown in Figure S2 (Supplementary Material). Cattle were most frequently observed ‘eating/drinking’ (29%) and ‘exploring bedding’ (26%) and infrequently observed walking (3% of observations). Engagement with environmental enrichment items was observed on 0.8% of observations and abnormal behaviours were never recorded.

QBAs were carried out on 29 occasions for one or more of six of the cattle (See Supplementary Material for components). On five occasions, additional enrichment in the form of a cardboard box was added to the pen. On one occasion, food enrichment was provided. Observers noted that they would like to provide more enrichment and/or human contact on 13 occasions. Only one animal had a positive ‘Mood’ score on every occasion. The mean recorded ‘Overall Mood’ score was 60 mm (range 12 mm–94 mm). At the end of each data collection session, staff ‘Willingness to take on the life the animal is now living’ (scale of 1–20) was recorded. Scores greater than 1 were only recorded on four occasions (three scores of 2, and one score of 3).

### 3.6. Goats

#### 3.6.1. Animal Population and Husbandry

Three 13-month-old castrated male British Saanen crossbred goats occupying the same building as the cattle as part of a scrapie transmission study but housed across two pens, each measuring 3.5 m × 7.8 m, connected via a corridor, were included in the study. Their provisions included straw and sawdust bedding and enrichment of rubber rings, a climbing platform, and browsing material. They were fed a commercial concentrate diet enriched with ammonium chloride to prevent urinary calculi.

#### 3.6.2. Data Collection

Data collection was conducted by the same staff member who assessed the cattle, and consisted of scan sample observations to provide behavioural time budgets against an ethogram (goats observed in turn every 15 s, for a total of 10 min); QBA score for 13 terms [17] on 120 mm visual analogue scales; overall mood score recorded on 120 mm visual analogue scale; willingness of assessor to take on the life of the animal (20-point scale: 1 = extremely unwilling, 20 = extremely willing); and opportunities for pleasure/suggestions for change between assessments. See further descriptions in the Supplementary Material (Goats—Welfare Assessment Protocols).

#### 3.6.3. Behavioural Observations

Behavioural observations of three goats in Unit C occurred on 24 occasions, and then of the two remaining goats on four occasions using the ethogram provided in Supplementary Information. On some occasions, individual animals or pens were missed due to demands of other working requirements. The most frequently observed behaviour was ‘standing ruminating’ (20.3% of observations), followed by ‘standing inactive’ (16.9%), ‘moving’ (14.1%), ‘lying ruminating’ (13.8%), and ‘lying inactive’ (10.9%). The most frequently observed use of physical enrichment was with the brush (1.1%), followed by the wheel (0.5%) and empty disinfectant drum (0.4%) (all present continuously), and finally the cardboard box, which was only given on two observation days (see Figure S3, Supplementary Material).

QBAs of one or more individual goats were conducted on 28 days (see Supplementary Information for components). The minimum ‘Overall Mood’ score recorded was 2 mm and the maximum was 92 mm, with a mean score of 48 mm. A score of the ‘Willingness to take on the life the animal is now living’ on a scale of 1 to 20 was recorded at the end of each session and a score greater than 1 was only recorded on two occasions (both scores = 2).

### 3.7. Calves and Sheep

The additional voluntary welfare assessment protocols for calves and sheep can be found in Supplementary Information but are not described here in detail as data were not collected. The sheep protocol was not assessed due to it taking an unfeasibly long time (>30 min) for the sheep to return to normal behaviour in the presence of an observer. The calf protocol was not assessed due to lack of sufficient staff time in that unit.

### 3.8. Staff Feedback

Due to staff changes, only two of the three staff who conducted assessments were available to respond to the feedback survey. One person ‘somewhat agreed’ and one person ‘completely agreed’ that the welfare protocol reflected staff ideas from the initial workshop was easy to implement and that there was sufficient time. Both staff ‘completely agreed’ that carrying out the assessments positively influenced how they worked with the animals under their care, that reviewing the data helped them to understand animal welfare needs, and that they would recommend the process to colleagues in similar institutes. In response to a question asking about the benefits they would tell a colleague about conducting the assessments, both respondents highlighted that the additional time spent with and observing the animals gave them a better insight into their individual personalities and behaviours which in turn made it easier to identify suitable enrichments and adapt their environment to suit them. In addition, one respondent felt that it was beneficial to be part of a process increasing the understanding of the welfare needs of experimental farmed animal species, which they felt were less well-understood than for mice and rats. Both staff members reported it was a challenge to find the time to complete the assessments, especially when busy or short-staffed. In addition, one respondent found building design sometimes impeded observations, and that ensuring other staff did not interrupt observations could be difficult. Both staff members thought it would be useful if more staff conducted the observations. Both staff members commented in a final ‘any other comments’ question that they had enjoyed the process.

## 4. Discussion

This innovative initiative provided a unique opportunity to explore a facilitated, co-creation approach to additional voluntary welfare assessment of farmed animal species in a research facility, with a particular focus on positive experiences for the animals. Harnessing the motivation of staff is essential for successful innovation, particularly in the public sector [18]. In our study staff who engaged fully with the process, being active in both developing the additional voluntary welfare assessment protocols and conducting observations, reported high levels of satisfaction with the process, considered it to be useful for understanding and engaging with the animals, and would recommend it to colleagues. However, of the nine staff members who participated in the initial workshop, only three took part in the welfare monitoring phase, and two provided feedback. This is a significant limitation of the study which reduces the generalisability of the findings. Information was not available as to the reasons why staff did not conduct assessments, but, whilst it was encouraged, it was not a mandatory requirement and thus, staff prioritised other activities as part of their challenging day job that included live animal sampling, general husbandry tasks, and standard welfare monitoring. Further, staff had already suggested that they had relatively few concerns about the welfare of the calves and sheep and therefore may not have felt welfare monitoring was likely to yield as many benefits. They also found that the sheep took a long time to return to normal behaviour and therefore, observations were particularly time-consuming. Revisiting the staff and trialling adjustments to the protocols may have facilitated some observations to take place.

Encouraging bespoke protocols was done to promote buy in of the process by staff, and it was recognised at the outset that this would likely lead to some compromise in quality of data collected. Given that the flexibility offered did not result in sufficient buy-in from staff to record robust data anyway, it might be useful in future to propose at the outset some core measures that could be harmonised across protocols and expanded in response to staff suggestions. It is interesting to note the slightly different approaches taken, featuring physical assessments to varying degrees between species, for example. However, this may have influenced implementation as, for example, in one pig unit, physical condition was only rated three times, providing a snapshot of physical welfare but little meaningful comparative data over time. Having one person per tested unit may have been beneficial for observation consistency but it highlights relatively poor uptake and engagement with the welfare monitoring programme across the initial cadre of nine. Furthermore, the assessments that were made by staff who engaged with the process were relatively sporadic and incomplete, and there appeared to be differences in what type of assessment staff were most able or willing to conduct. Overall, the scan sampling behavioural observations appear to have been the most successfully implemented welfare assessment technique across all units, with staff conducting more of these than any other observations. Furthermore, assessments of ‘Overall Mood’ and staff ‘Willingness to live the lives of the animals under their care’ appear to have been more accessible than creating the QBA scores. Another study of QBA revealed that participation is more valuable than the data produced, where engagement with the process led to meaningful human behaviour change [19]. The exact format of the protocol for each measure may influence the willingness to conduct the assessment, as well as the reliability of the results. For example, the 1–20 scales employed for evaluating overall mood, and pig response to training were only anchored by descriptors at 1 and 20, and additional anchoring, especially around the mid-point, may be useful. Further, the importance of training protocols tailored to individual animals’ needs is essential [20,21] and could be captured through such a monitoring system.

With staff feedback of the overall experience provided by only two members of staff who had completed the process, it was not possible to gain a full picture of the direct barriers experienced by individuals who did not participate in the welfare assessments. It is likely that there was a self-selection bias in that staff who conducted assessments and that provided feedback were already more positively disposed towards animal welfare and therefore motivated to conduct assessments and report positive feedback. It may be useful for institutions to harness the intrinsic motivation of animal welfare orientated staff to promote successful implementation of such welfare monitoring, for example, through assigning specific responsibilities in this regard. In addition, it is not known how any recall bias given the length of time (more than a year) between conducting assessments and providing feedback may have affected their responses. However, perceived lack of sufficient time was identified as an issue, alongside needing more support from co-workers to conduct uninterrupted observations, and improvement of visual access to the animals. Despite the additional voluntary welfare assessment protocols taking <20 min each, this suggests that institutions could support their workers so that animal welfare observations can be conducted as part of work duties, with regular scheduling into daily or weekly work plans, and regular reporting of findings.

The welfare assessments were not continued after the exploratory programme ended, and it is likely that the process could be further developed to encourage longer-term staff engagement. For example, employee screening to identify workers who would be most willing to engage in welfare monitoring as part of their responsibilities may maximise uptake and optimise opportunities for human behaviour change for improved animal husbandry. It may also boost the sense of job satisfaction for workers who wish to engage in monitoring and improving the lives of the animals in their care through driving change in working practice.

Although the results of the assessments should not be overinterpreted due to the sporadic nature of the observations, they nevertheless provide an insight into welfare at the unit. In addition, a more systematic application of any protocol and regular standardised assessments would enhance the quality of the data with which management decisions can be made to improve welfare. The findings should not be generalised more widely, but nevertheless serve the illustrative point that, for example, there were clear differences between the behavioural time budgets recorded of pigs in the two settings [19], illustrating a potential use for such comparison data within a single organisation. Further, if appropriately designed, behavioural observations can provide a comparison of time budgets with those of animals in semi-wild or less restricted settings and insights into aspects that could be a focus for welfare improvements [22,23,24]. Cattle engagement with environmental enrichment was rarely recorded. Given the infrequent nature of these behaviours, their occurrence may be difficult to capture in short scan samples, but environmental enrichment may still provide the animals with significant positive experiences. Importantly, observations of lack of enrichment use frequently triggered the staff to provide novel enrichments, in a good example of utilising observations to drive welfare improvements [25]. It was interesting that staff scored ‘Overall Mood’ for cattle and goats as approximately midway on the visual analogue scale, despite their ‘Willingness to live the lives of the animals in their care’ being very low. The very low ‘Willingness to live the lives of the animals in their care’ scores of 1 or 2 (out of 20) are of concern and staff had expressed specific concerns about these animals at the outset. Institutions could collect these data to form the basis for informal staff discussions to encourage welfare improvement strategies and more formally could be used to trigger a welfare intervention process when below a certain threshold. In addition, this type of information could inform the severity bandings ascribed to animals and/or procedures or the requirements related to the bandings as it may provide greater insight into societal views on the lives of such animals.

Overcoming the significant challenges experienced in this study in continuing staff engagement beyond the protocol development through to the data collection would likely require a range of strategies. Systems that make the data collection easier, or more automated, may help to reduce the time burden associated with additional welfare monitoring. Such systems are advancing fast for laboratory rodents, for example, in utilising facial expressions and body posture [26], and for farmed animals, for example, to recognise tail biting in pigs [27] or play behaviour in dairy calves [28]. Sensors and video recordings are increasingly used to study animal behaviour in large animals [29], although limited sensor technology currently exists for pigs or goats due to their explorative and destructive behaviour, and analysis of video recordings by machine learning or sensor data is limited to specific behaviours, such as lying, standing, eating, etc., and would not inform mood or overall welfare, which the welfare assessments tried to address. There is also a lack of validation and precision of these techniques [30], particularly in confined spaces applicable to research animals, which is why they cannot currently replace human observations. Even if the time to conduct assessments were reduced, institutions will also need to embed the culture of such monitoring within their policies and practices, ensuring that monitoring is integral to staff roles, that analysis and reporting forms part of the process, and that staff are encouraged and empowered to be creative in finding positive welfare solutions. Finally, innovations to improve animal welfare should be tracked not just in the short term, as in this study, but in the longer term to be able to fully evaluate the sustained impact of such interventions.

## 5. Conclusions

Overall, our results provide insight for farm animal institutions wanting to implement innovative welfare assessment methods in the future, focusing on those aspects that appear most feasibly applicable in situ. Our findings suggest that initial co-creation and engagement, although important conditions for successful innovations, may be insufficient for full implementation of voluntary welfare monitoring, and prolonged support or other mechanisms are likely needed to fully embed the process. This could include allowing dedicated time to conduct additional welfare assessments, regular reporting of findings internally, identifying and rewarding welfare monitoring ‘champions’ within an organisation, and providing further training for staff. In addition, training and standardisation of staff and protocols would be beneficial to ensure that data collected are robust and valid, especially if they are being used as more than a prompt to promote human behaviour changes to improve welfare (for example, to add enrichments when observations suggest they may be beneficial), and instead to track welfare over time, or comparing welfare in different units. As technological advances progress, it may be that automating some elements of welfare monitoring may help to reduce the human resource burden.

## Data Availability

The data presented in this study are available on request from the corresponding author due to confidentiality reasons.

## Supplementary Materials

The following supporting information can be downloaded at: https://www.mdpi.com/article/10.3390/ani15192817/s1, Figure S1. The proportion of observations that pigs spent performing each behaviour (rest; positive social behaviour; negative social behaviour; exploratory behaviour towards manipulable material/object; exploratory behaviour towards pen fixtures; eating/drinking; play; other) during observations at Unit B (n = 15 pigs, 70 observation sessions) and Unit A (n = 4 pigs, 23 observation sessions). Figure S2. The proportion of observations that cattle (n = 8) were observed performing each behaviour during observation sessions (n = 27). Figure S3. The proportion of observations that goats (n = 3) spent performing each behaviour (ruminating standing; standing inactive; moving; ruminating lying; lying inactive; eating/drinking/explore pen/bedding/self-groom; enrichment: brush; salt lick; positive social; enrichment: wheel; enrichment: empty disinfectant drum; play; negative social; approaching observer; enrichment: cardboard box; standing/approaching observer; abnormal behaviour; enrichment: chain licking; out of sight; other) during observations (n = 28 observation sessions).
