# Supplementary material for: Voluntary Additional Welfare Monitoring of Farm Animals Used in Research: Maximising Benefits Requires Sustained Support
Source: Animals (Basel). 2025 Sep 26;15(19):2817. doi: 10.3390/ani15192817 (PMC12524281; doi:10.3390/ani15192817)
Supplement: Supplementary file 1 [file animals-15-02817-s001.zip › animals-3775005-supplementary.pdf]

Supplementary material: welfare assessment protocols, staff feedback questions and QBA PCA results

Pigs- Welfare Assessment Protocol

**Lameness**		
**Observation**		Observe each pig walk a few steps from behind and the side, ideally on an even surface
**Scoring**	**0**	Normal gait; long, fluid, even strides; minimal 'swagger' of the hind end
	**1**	Uneven gait, shortened strides, hind end swagger, likely identify an affected limb
	**2**	Minimal weight-bearing on the affected limb whilst standing and when moving
	**3**	No weight bearing on the affected limb
**Record**		Lameness score for each pig

**Skin condition**		
**Observation**		Observe the condition of the skin that can be seen in at least a 10cm x 10cm area
**Scoring**	**0**	No skin abnormality
	**1**	Skin is flaky/dry
	**2**	Skin is scabby/pink
	**3**	Cracks or open sore areas are present
**Record**		The maximum score seen in any area of skin at least 10cm x 10cm

**Body marks**		
**Observation**		Stand near the pig and observe one side only
**Scoring**	**0**	No lesions or less than mild lesions as described below
	**1**	A skin-depth linear lesion longer than 10cm<br>OR 3 or more 3cm lesions<br>OR a circular area larger than 1cm diameter
	**2**	Lesion or area of lesions ≥ 5x5cm diameter<br>OR the lesions cover a large percentage (>25%) of the skin<br>OR lesion that extends >0.5cm into deeper layers of tissue
	**3**	>1cm deep lesion at least 3cm long
**Body Regions**		**Ears and head (E)**<br>**Shoulder (S)**<br>**Flank (F)**<br>**Hindquarters (H)**<br>**Legs (L)**
**Record**		The score for each body region for each pig

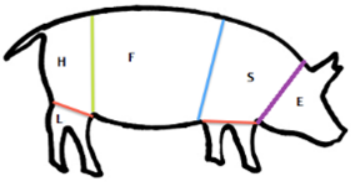

**Neck skin thickness**		
**Observation**		Use calipers to record the skin thickness over the jugular vein
**Record**		The caliper reading (mm)

Weight tape

Observation Use the weight tape on sows during handling

Record The girth beind the elbow (cm)
The length of the body (cm)
The estimated weight of the sow (kg)

Body condition

Observation Visually assess from the side and behind, using manual palpation when possible

Scoring

Thin Score 1 or 2: Ribs, backbone, 'H' bones and 'pin' bones obvious (or easily detected with pressure)

Moderate Score 3: Ribs, backbone, 'H' bones and 'pin' bones barely visible (or barely felt with firm pressure)

Fat Score 4 or 5: Ribs, backbone, 'H' bones and 'pin' bones cannot be seen (or felt even when pressure is applied) or fats deposits are clearly visible

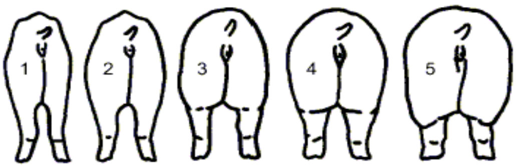

Record Thin (T), Moderate (M) or Fat (F) for each sow

Handling Stress

Observation Observe each pig during handling procedures

Scoring

0 Normal skin colouration, no vocalisation
1 Nose and/or tip of ears red/purple. May vocalise at this stage
2 Red blotchy colour extended beyond the nose/tip of ears. Likely to vocalise
3 Red blotchy colour extends to at least 75% of the body. Likely to vocalise
4 Body has turned purple/blue, no vocalisation

Record Handling stress score for each pig

Behavioural observations

Observation Observe all the pigs in turn, every 15 seconds, for a total of 10 minutes. Ideally conducted at approximately the same time of day

Ethogram

Resting lying not engaged in other activities, may be asleep
Positive social behaviour sniffing/nosing/licking/moving gently away. No flight reaction
Negative social behaviour aggressive or other act that elicits a response from the other animal
Exploratory behaviour towards manipulable material/object sniffing/nosing/licking/rooting clean manipulable materials such as straw or earth
Exploratory behaviour toward pen fixtures sniffing/nosing/licking pen fixtures
Eating/drinking eating/drinking not associated with exploratory behaviour
Play any form of social/locomotor or object play
Other any other behaviour

Record Tally mark in each behaviour category for each pig

Mood score		
**Observation**		Following the behavioural observations make a qualitative assessment of each pig
**Scoring**		Rate each pig on a 20 point scale for their mood
	**1**	the worst mood a pig could experience- e.g. severe pain, apathy, fear
	**20**	the best mood a pig could experience- e.g. excitement, great contentment, happily busy
**Record**		Circle a number from 1 to 20

Response to training		
**Observation**		Following a training session make an assessment of the demeanour of each pig
**Scoring**		Rate each pig on a 20 point scale for their demeanour during training
	**1**	the worst experience a pig could have- frustrating, fearful
	**20**	the best experience a pig could have- highly enjoyable, exciting, pleasurable
**Record**		Circle a number from 1 to 20

Pigs- Welfare Assessment

Observer ______ Date ______ Time ______

Sow ID ______ Lameness 0/1/2/3 ______ Body condition T/M/F ______

Body Marks 0/1/2/3

Ears/head	Shoulders	Flank	Hindquarters	Legs

Skin thickness (mm) ______

Weight tape

girth (cm)	length (cm)	weight (kg)

Handling stress 0/1/2/3/4 ______

Response to training

Skin Condition 0/1/2/3 ______

Extremely negative — Extremely positive

1 2 3 4 5 6 7 8 9 10 11 12 13 14 15 16 17 18 19 20

Sow ID ______ Lameness 0/1/2/3 ______ Body condition T/M/F ______

Body Marks 0/1/2/3

Ears/head	Shoulders	Flank	Hindquarters	Legs

Skin thickness (mm) ______

Weight tape

girth (cm)	length (cm)	weight (kg)

Handling stress 0/1/2/3/4 ______

Response to training

Skin Condition 0/1/2/3 ______

Extremely negative — Extremely positive

1 2 3 4 5 6 7 8 9 10 11 12 13 14 15 16 17 18 19 20

Sow ID ______ Lameness 0/1/2/3 ______ Body condition T/M/F ______

Body Marks 0/1/2/3

Ears/head	Shoulders	Flank	Hindquarters	Legs

Skin thickness (mm) ______

Weight tape

girth (cm)	length (cm)	weight (kg)

Handling stress 0/1/2/3/4 ______

Response to training

Skin Condition 0/1/2/3 ______

Extremely negative — Extremely positive

1 2 3 4 5 6 7 8 9 10 11 12 13 14 15 16 17 18 19 20

Comments:

Sow ID | | Lameness 0/1/2/3 | | Body condition T/M/F | |

	Ears/head	Shoulders	Flank	Hindquarters	Legs		
Body Marks 0/1/2/3						Skin thickness (mm)	

	girth (cm)	length (cm)	weight (kg)		
Weight tape				Handling stress 0/1/2/3/4	

Response to training Skin Condition 0/1/2/3 | |

Extremely negative — Extremely positive

1	2	3	4	5	6	7	8	9	10	11	12	13	14	15	16	17	18	19	20

Sow ID | | Lameness 0/1/2/3 | | Body condition T/M/F | |

	Ears/head	Shoulders	Flank	Hindquarters	Legs		
Body Marks 0/1/2/3						Skin thickness (mm)	

	girth (cm)	length (cm)	weight (kg)		
Weight tape				Handling stress 0/1/2/3/4	

Response to training Skin Condition 0/1/2/3 | |

Extremely negative — Extremely positive

1	2	3	4	5	6	7	8	9	10	11	12	13	14	15	16	17	18	19	20

Sow ID | | Lameness 0/1/2/3 | | Body condition T/M/F | |

	Ears/head	Shoulders	Flank	Hindquarters	Legs		
Body Marks 0/1/2/3						Skin thickness (mm)	

	girth (cm)	length (cm)	weight (kg)		
Weight tape				Handling stress 0/1/2/3/4	

Response to training Skin Condition 0/1/2/3 | |

Extremely unpleasant — Extremely enjoyable

1	2	3	4	5	6	7	8	9	10	11	12	13	14	15	16	17	18	19	20

Comments:

Pigs- Behavioural observations

Observer		Date		Time		No. of pigs	

Scan	Rest	Pos social	Neg social	Expl manip	Expl pen	Eat/drink	Play	Other
Start								
15								
30								
45								
1 min								
15								
30								
45								
2 min								
15								
30								
45								
3 min								
15								
30								
45								
4 min								
15								
30								
45								
5 min								
15								
30								
45								
6 min								
15								
30								
45								
7 min								
15								
30								
45								
8 min								
15								
30								
45								
9 min								
15								
30								
45								
10 min								
Totals								

Mood score Sow ID

worst mood best mood

1	2	3	4	5	6	7	8	9	10	11	12	13	14	15	16	17	18	19	20

Mood score Sow ID

worst mood best mood

1	2	3	4	5	6	7	8	9	10	11	12	13	14	15	16	17	18	19	20

Mood score Sow ID

worst mood best mood

1	2	3	4	5	6	7	8	9	10	11	12	13	14	15	16	17	18	19	20

Mood score Sow ID

worst mood best mood

1	2	3	4	5	6	7	8	9	10	11	12	13	14	15	16	17	18	19	20

Mood score Sow ID

worst mood best mood

1	2	3	4	5	6	7	8	9	10	11	12	13	14	15	16	17	18	19	20

Opportunities for pleasure since last observation:

Any suggestions for changes:

Any other comments:

Unit C cattle- Welfare Assessment Protocol

Behavioural observations

Observation Observe all the cattle in turn, every 15 seconds, for a total of 10 minutes. Systematically change the time and day according to the pre-determined schedule in order to build up an overall picture of the activity of the animals.

Ethogram

**Lying inactive**	Lying not engaged in other activities, may be asleep
**Standing inactive**	Standing not engaged in other activites
**Observer**	Either standing at, or approaching, the observer (door)
**Ruminating standing**	Rhythmic chewing without ingesting whilst standing
**Ruminating lying**	Rhythmic chewing without ingesting whilst lying
**Moving**	Walking or faster movement
**Eating/drinking**	When no exploratory behaviour is also performed
**Use of enrichment (state type)**	Using enrichment- e.g brush/ investigatory box
**Exploratory behaviour toward pen fixtures**	sniffing/nosing/licking pen fixtures
**Exploratory behaviour toward bedding**	sniffing/nosing/licking bedding
**Positive social behaviour**	Social licking; head play- playful touching headhorn base/neck where neither takes advantage of the other
**Negative social behaviour**	Head butt- forceful; chasing- aggressive; fighting; displacement
**Self-grooming**	Licking or scratching any part of own body
**Play**	Any form of social, locomotor or object play
**Abnormal behaviour**	Non-functional behaviours. May be repetitive (stereotypic) including oral (e.g. tongue rolling) or locomotor (e.g. pacing)
**Other**	Any other behaviour
**Out of sight**	Unable to be viewed

Record Tally mark in each behaviour category. Use a new sheet for each animal

Qualitative behavioural Assessment

Observation Following the behavioural observations make a qualitative assessment of **each animal**
If it has not been possible to get an adequate assessment of each animal then spend additional time observing each pen before performing the qualitative assessment.
If you have insufficient time to observe all cattle in the unit just focus on one or two pens.

Scoring Consider HOW the animal is, rather than what they have been doing. Rate them against each of the qualities
In addition, rate each animal on a 20 point scale for their overall mood from:
a) the worst mood cattle could experience- e.g. severe pain, apathy, fear TO
b) the best mood cattle could experience- e.g. excitement, great contentment, happily busy

Record A mark on each line for each quality.

Unit C cattle - Qualitative behavioural assessment

Observer | Date | Time | Animal | Pen

Active

Min. Max.

Relaxed

Min. Max.

Fearful

Min. Max.

Agitated

Min. Max.

Calm

Min. Max.

Content

Min. Max.

Indifferent

Min. Max.

Frustrated

Min. Max.

Friendly to people

Min. Max.

Bored

Min. Max.

Playful

Min. Max.

Positively occupied

Min. Max.

Lively

Min. Max.

Inquisitive

**Irritable**	Min.	Max.
**Uneasy**	Min.	Max.
**Sociable to cattle**	Min.	Max.
**Apathetic**	Min.	Max.
**Happy**	Min.	Max.
**Distressed**	Min.	Max.

**Overall mood**	worst	Best

How willing would you be to take on the life this animal is living?

Extremely unwilling — Extremely willing

1	2	3	4	5	6	7	8	9	10	11	12	13	14	15	16	17	18	19	20

Opportunities for pleasure since last observation:

Any notable stressors since last observation?

Any suggestions for changes:

Any other comments:

Unit C cattle - Behavioural observations

Observer: ____________

Date: ____________

Time: ____________

ID of animals in pen 1			
ID of animals in pen 2			
ID of animals in pen 3			
ID of animals in pen 4			

Scan		Lying inactive	Standing inactive	Observer	Ruminating standing	Ruminating lying	Approaching observer	Moving	Eating/drinking	Enrichment……………	Enrichment……………	Enrichment……………	Enrichment……………	Enrichment……………	Enrichment……………	Explore pen	Explore bedding	Positive social	Negative social	Self-groom	Play	Abnormal behaviour	Other	Out of sight
1	Pen 1																							
	Pen 2																							
	Pen 3																							
	Pen 4																							
2	Pen 1																							
	Pen 2																							
	Pen 3																							
	Pen 4																							
3	Pen 1																							
	Pen 2																							
	Pen 3																							
	Pen 4																							
4	Pen 1																							
	Pen 2																							
	Pen 3																							
	Pen 4																							
5	Pen 1																							
	Pen 2																							
	Pen 3																							
	Pen 4																							

Time

Scan		Lying inactive	Standing inactive	Standing/approaching door	Ruminating standing	Ruminating lying	Approaching observer	Moving	Eating/drinking	Enrichment………….	Enrichment………….	Enrichment………….	Enrichment………….	Enrichment………….	Enrichment………….	Explore pen	Explore bedding	Positive social	Negative social	Self-groom	Play	Abnormal behaviour	Other	Out of sight
6	Pen 1																							
	Pen 2																							
	Pen 3																							
	Pen 4																							
7	Pen 1																							
	Pen 2																							
	Pen 3																							
	Pen 4																							
8	Pen 1																							
	Pen 2																							
	Pen 3																							
	Pen 4																							
9	Pen 1																							
	Pen 2																							
	Pen 3																							
	Pen 4																							
10	Pen 1																							
	Pen 2																							
	Pen 3																							
	Pen 4																							

Total pen 1																							
Total pen 2																							
Total pen 3																							
Total pen 4																							

Goats- Welfare Assessment Protocol

Behavioural observations

Observation Observe all the goats in turn, every 15 seconds, for a total of 10 minutes. Systematically change the time and day according to the pre-determined schedule in order to build up an overall picture of the activity of the animals.

Ethogram

**Lying inactive**	Lying not engaged in other activities, may be asleep
**Standing inactive**	Standing not engaged in other activites
**Observer**	Approaching/ staring at observer
**Ruminating standing**	Rhythmic chewing without ingesting whilst standing
**Ruminating lying**	Rhythmic chewing without ingesting whilst lying
**Moving**	Walking or faster movement
**Eating/drinking**	When no exploratory behaviour is also performed
**Use of enrichment (state type)**	Using enrichment- e.g brush/ investigatory box
**Exploratory behaviour toward pen fixtures**	sniffing/nosing/licking pen fixtures
**Positive social behaviour**	Social licking; head play- playful touching headhorn base/neck where neither takes advantage of the other
**Negative social behaviour**	Head butt- forceful; chasing- aggressive; fighting; displacement
**Self-grooming**	Licking or scratching any part of own body
**Play**	Any form of social, locomotor or object play
**Abnormal behaviour**	Non-functional behaviours. May be repetitive (stereotypic) including oral (e.g. tongue rolling) or locomotor (e.g. pacing)
**Other**	Any other behaviour
**Out of sight**	Unable to be viewed

Record Tally mark in each behaviour category. Use a new sheet for each animal

Qualitative behavioural Assessment

Observation Following the behavioural observations make a qualitative assessment of **each animal**
If it has not been possible to get an adequate assessment of each animal then spend additional time observing each goat before performing the qualitative assessment.

Scoring Consider HOW the animal is, rather than what they have been doing. Rate them against each of the qualities
In addition, rate each animal on a 20 point scale for their overall mood from:
a) the worst mood goats could experience- e.g. severe pain, apathy, fear TO
b) the best mood goats could experience- e.g. excitement, great contentment, happily busy

Record A mark on each line for each quality.

Goat - Qualitative behavioural assessment

Observer | Date | Time | Animal

Aggressive

Min. Max.

Agitated

Min. Max.

Alert

Min. Max.

Bored

Min. Max.

Content

Min. Max.

Curious

Min. Max.

Fearful

Min. Max.

Frustrated

Min. Max.

Irritated

Min. Max.

Lively

Min. Max.

Relaxed

Min. Max.

Sociable

Min. Max.

Suffering

Overall

worst	Best
├──────────────────┤	

How willing would you be to take on the life this animal is living?

Extremely unwilling — Extremely willing

1	2	3	4	5	6	7	8	9	10	11	12	13	14	15	16	17	18	19	20

Opportunities for pleasure since last observation:

Any notable stressors since last observation?

Any suggestions for changes:

Any other comments:

Goat- Behavioural observations

Observer

Date Time

Scan	Lying inactive	Standing inactive	Ruminating standing	Ruminating lying	Moving	Eating/drinking	Use of enrichment 1	Use of enrichment 2	Use of enrichment 3	Explore pen	Positive social	Negative social	Self-groom	Play	Abnormal behaviour	Other
Start																
15																
30																
45																
1 min																
15																
30																
45																
2 min																
15																
30																
45																
3 min																
15																
30																
45																
4 min																
15																
30																
45																
5 min																
15																
30																
45																
6 min																
15																
30																
45																
7 min																
15																
30																
45																
8 min																
15																
30																
45																
9 min																
15																
30																
45																
10 min																
Totals																

Calves protocol

Behavioural observations

Observation Observe the cattle for a total of 10 minutes within 1 hour of new feed being provided.

Ethogram A new bout is recorded if there is a 10 second pause between bouts

**Head Butt**	Physical pushing of head onto another animal (no displacement)
**Displacement**	Any type of physical contact that results on another animal moving
**Chasing**	chaser makes another animal flee by fast or agitated movements
**Fighting**	Two contestants pushing vigorously
**Forced rising**	Physical contact that makes another animal rise
**Social licking**	Licks any part of the body of another animal
**Horn/head play**	Mutual head play with physical contact but no negative response
**Play**	Any form of locomotor, object or social play

Record Tally mark for each occurance of behaviour observed in the 10 minutes

Coughing

Observation Observe the cattle for a total of 10 minutes within 1 hour of new feed being provided.

Scoring A coughing bout from one animal is heard. A new bout is recorded with each new animal that coughs, and if the same animal has at least a 10 second break between coughs.

Record Tally mark for each coughing bout observed in the 10 minutes

Qualitative behavioural assessment (QBA)

Observation Following the behavioural observations make a qualitative assessment of the group
If it has not been possible to get an adequate assessment of the group then spend additional time observing before performing the qualitative assessment.

Scoring Consider HOW the animal is, rather than what they have been doing. Rate them against each of the qualities
In addition, rate each animal on a 20 point scale for their mood

1 worst mood cattle could experience- e.g. severe pain, apathy, fear
20 best mood cattle could experience- e.g. excitement, great contentment, happily busy

Record A mark on each line for each quality.
Circle a number from 1 to 20 representing the mood of that group.

Level of social integration

Observation Observe the cattle both formally for 10 minutes and informally during routine care

1 cattle are in clear familiar groups, not uniformly spaced out
20 cattle are completely integrated across mixed groups

Record Circle a number from 1 to 20 relating to level of social integration

Calves recording sheet

Observer				Date		Days since arrival		No. in group	
Observed	housing	at pasture	circle	Time					

		Record a tally for each occurance in 10 minutes	Total
Antagonistice behaviours	Head Butt		
	Displacement		
	Chasing		
	Fighting		
	Enforced rising		
Cohesive behaviours	Social licking		
	Horn/head play		
	Play		
	Coughs		

Calves - Qualitative behavioural assessment

Observer		Date		Days since arrival		No. in group	
Observed	housing / at pasture / circle	Time					

**Active**	Min.	Max.
**Relaxed**	Min.	Max.
**Uncomfortable**	Min.	Max.
**Calm**	Min.	Max.
**Content**	Min.	Max.
**Tense**	Min.	Max.
**Enjoying**	Min.	Max.
**Indifferent**	Min.	Max.
**Frustrated**	Min.	Max.
**Friendly to humans**	Min.	Max.
**Bored**	Min.	Max.
**Positively occupied**	Min.	Max.
**Inquisitive**	Min.	Max.
**Irritable**	Min.	Max.
**Nervous**	Min.	Max.

**Boisterous**	Min.	Max.
**Uneasy**	Min.	Max.
**Sociable to animals**	Min.	Max.
**Happy**	Min.	Max.
**Distressed**	Min.	Max.

Overall

worst mood																			best mood
1	2	3	4	5	6	7	8	9	10	11	12	13	14	15	16	17	18	19	20

Level of social integration

groups separated																			groups totally integrated
1	2	3	4	5	6	7	8	9	10	11	12	13	14	15	16	17	18	19	20

Any enrichments provided?

Any suggestions for changes:

Any other comments:

Sheep Welfare Assessment Protocol

Behavioural observations

Observation Observe all the sheep every 30 seconds, for a total of 10 minutes. Count how many sheep are doing each behaviour in turn.

Ethogram

**Lying**	Lying, may be resting or ruminating or may be asleep
**Standing**	Standing inactive or ruminating
**Eating……….**	Eating (state food available)
**Using enrichment**	engaging in enrichment or observing other using enrichment
**Exploratory behaviour toward pen fixtures**	sniffing/nosing/licking pen fixtures
**Social behaviour**	either positive or negative close interaction with another sheep
**Play**	Any form of social, locomotor or object play
**Other**	Any other behaviour, including walking, grooming, drinking

Record Tally mark in each behaviour category. Use a new sheet for each session

Enrichment use observations

Observation Opportunistically observe the sheep and count how many are using enrichment(s)
Complete these observations interspersed in daily routines when sheep behaviour not disturbed by observer's presence

Record The number of sheep using each enrichment(s)

Qualitative behavioural Assessment

Observation Following the behavioural observations make a qualitative assessment of the group
If it has not been possible to get an adequate assessment of the group then spend additional time observing before performing the qualitative assessment.

Scoring Consider HOW the animal is, rather than what they have been doing. Rate them against each of the qualities
In addition, rate each animal on a 20 point scale for their mood

1 worst mood sheep could experience- e.g. severe pain, apathy, fear

20 best mood sheep could experience- e.g. excitement, great contentment, happily busy

Record A mark on each line for each quality.
Circle a number from 1 to 20 representing the mood of that group.

Sheep- Qualitative behavioural assessment

Observer | Date | Time | Group

**Alert**		
	Min.	Max.
**Active**		
	Min.	Max.
**Relaxed**		
	Min.	Max.
**Fearful**		
	Min.	Max.
**Content**		
	Min.	Max.
**Agitated**		
	Min.	Max.
**Sociable**		
	Min.	Max.
**Aggressive**		
	Min.	Max.
**Vigorous**		
	Min.	Max.
**Subdued**		
	Min.	Max.
**Physically uncomfortable**		
	Min.	Max.
**Defensive**		
	Min.	Max.
**Calm**		
	Min.	Max.
**Frustrated**		
	Min.	Max.
**Apathetic**		

**Wary**	Min.	Max.
**Tense**	Min.	Max.
**Bright**	Min.	Max.
**Inquisitive**	Min.	Max.
**Assertive**	Min.	Max.
**Listless**	Min.	Max.

Overall

worst mood — best mood

1	2	3	4	5	6	7	8	9	10	11	12	13	14	15	16	17	18	19	20

Opportunities for pleasure since last observation:

Any notable stressors since last observation?

Any suggestions for changes:

Any other comments:

Sheep- Behavioural observations

Observer

Date Time No in group

Scan	Lying	Standing	Eating	Eating	Enrichment..........	Enrichment..........	Enrichment..........	Explore pen	Social behaviour	Play	Other
Start											
30											
1 min											
30											
2 min											
30											
3 min											
30											
4 min											
30											
5 min											
30											
6 min											
30											
7 min											
30											
8 min											
30											
9 min											
30											

10 min

Totals											

Staff feedback questions

1) Thinking about the process as a whole, please rate the following statements:

(completely disagree; somewhat disagree; neither agree nor disagree; somewhat agree; completely agree)

   a) The protocol reflected my ideas in the workshop
   b) The assessments were easy to carry out
   c) I had enough time to conduct the assessments
   d) Doing the assessments positively influenced how I worked with the animals
   e) Reviewing the data is useful in understanding the welfare of the animals
   f) I would recommend developing and conducting similar welfare assessments to a colleague in a similar position

2) Imagine a colleague of yours at a similar institute asks whether they should get involved in an equivalent initiative. What would you say would be the positives about doing so?
3) And what would you tell your colleague might be some of the challenges?
4) Please provide any comments on how any part of the process could be improved.
5) Do you have any other comments?

Pig Assessment Results

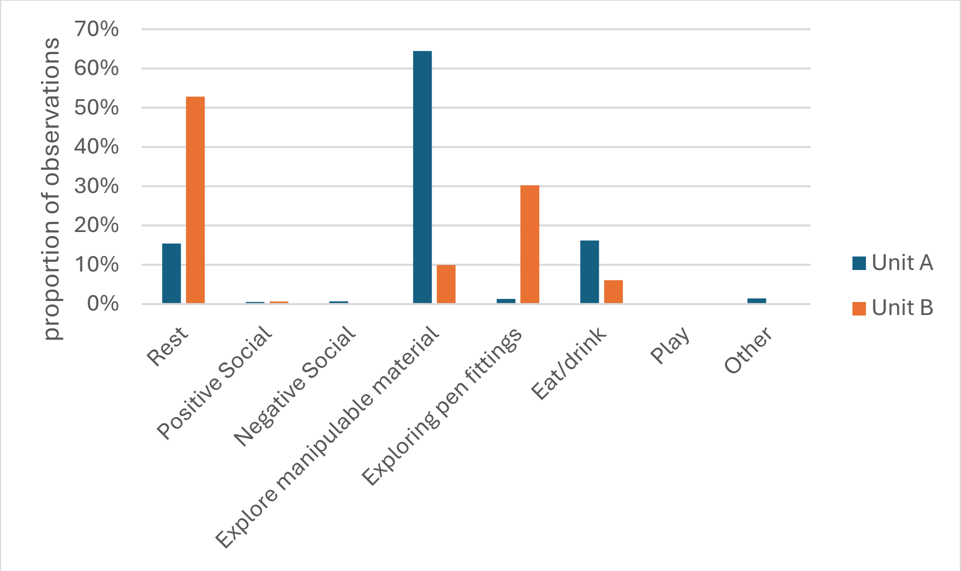

Figure S1 The proportion of observations that pigs spent performing each behaviour (rest; positive social behaviour; negative social behaviour; exploratory behaviour towards manipulable material / object; exploratory behaviour towards pen fixtures; eating/drinking; play; other) during observations at Unit B (n=15 pigs, 70 observation sessions) and Unit A (n=4 pigs, 23 observation sessions).

Cattle assessment results

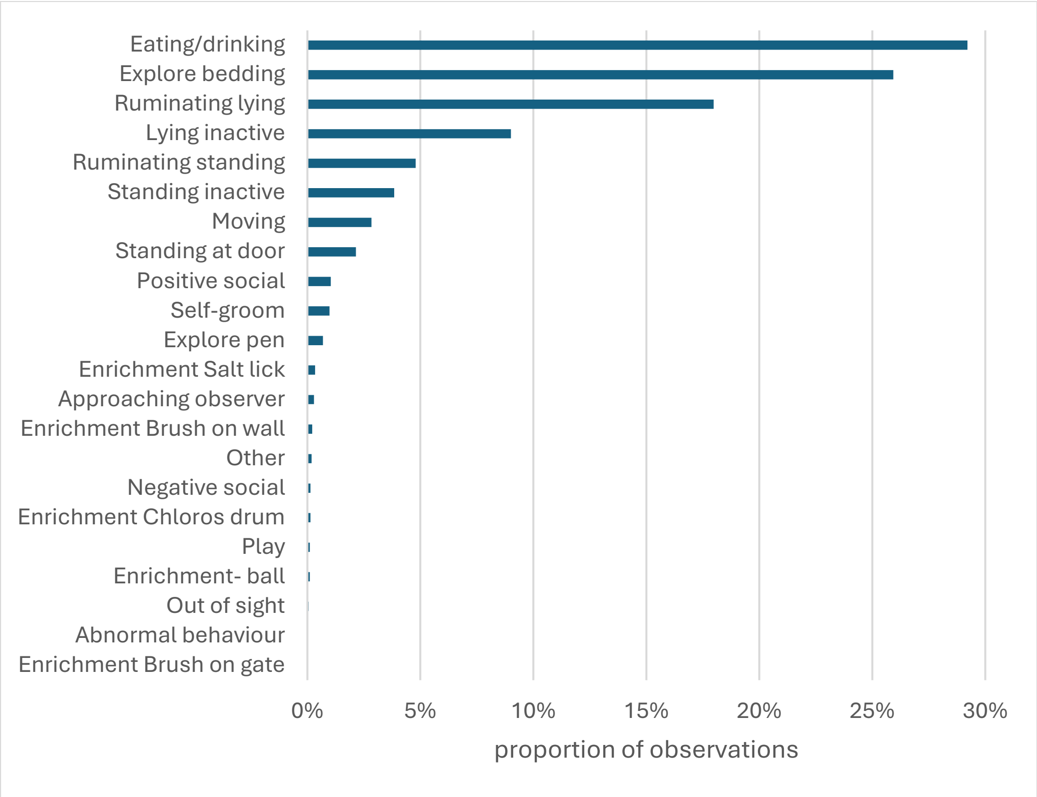

Figure S2. The proportion of observations that cattle (n=8) were observed performing each behaviour during observation sessions (n=27).

QBA results

Principal component analysis (PCA, covariance matrix, no rotation) was conducted in SPSS vs27 on QBA term scores to derive the loadings of each term on the major components.

Total Variance Explained

Component		Initial Eigenvalues[a] Total	% of Variance	Cumulative %	Extraction Sums of Squared Loadings Total	% of Variance	Cumulative %
Raw	1	3644.538	29.157	29.157	3644.538	29.157	29.157
	2	2385.961	19.088	48.245	2385.961	19.088	48.245
	3	1233.520	9.868	58.114	1233.520	9.868	58.114
	4	1051.159	8.410	66.523	1051.159	8.410	66.523
	5	886.462	7.092	73.615	886.462	7.092	73.615
	6	662.765	5.302	78.918	662.765	5.302	78.918
	7	508.307	4.067	82.984			
	8	397.163	3.177	86.162			
	9	358.063	2.865	89.026			
	10	297.368	2.379	91.405			
	11	244.971	1.960	93.365			
	12	182.663	1.461	94.826			
	13	151.090	1.209	96.035			
	14	131.825	1.055	97.090			
	15	104.842	0.839	97.929			
	16	82.781	0.662	98.591			
	17	70.695	0.566	99.156			
	18	50.608	0.405	99.561			
	19	39.315	0.315	99.876			
	20	15.528	0.124	100.000			

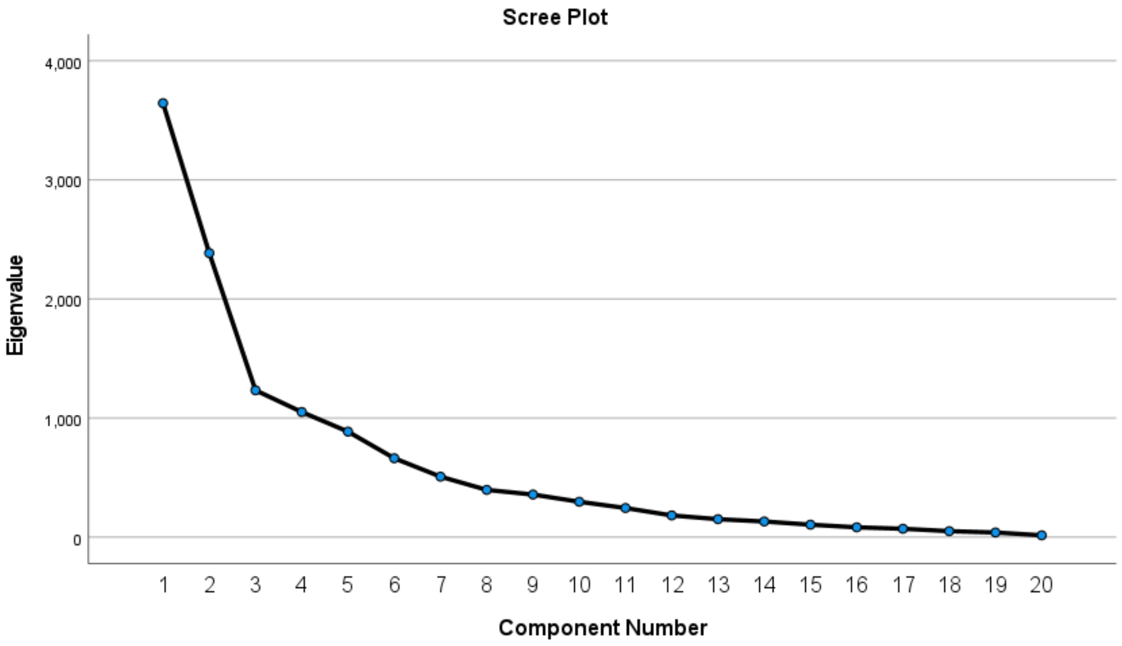

Loadings of terms on each component

Component 1	Component 2	Component 3

Playful	0.816	Uneasy	0.651	Friendly	0.766
Active	0.808	Agitated	0.569	Sociable	0.661
Inquisitive	0.775	Indifferent	0.541	Bored	0.291
Lively	0.749	Bored	0.465	Playful	0.275
Positively occupied	0.631	Fearful	0.458	Fearful	0.240
Irritable	0.617	Frustrated	0.377	Active	0.198
Agitated	0.542	Irritable	0.321	Inquisitive	0.197
Frustrated	0.489	Distressed	0.299	Lively	0.182
Uneasy	0.351	Apathetic	0.182	Indifferent	0.152
Happy	0.339	Playful	0.085	Positively occupied	0.076
Relaxed	0.289	Inquisitive	-0.015	Happy	0.039
Bored	0.085	Sociable	-0.032	Relaxed	0.019
Fearful	0.013	Happy	-0.077	Distressed	-0.010
Indifferent	-0.005	Lively	-0.079	Calm	-0.013
Content	-0.071	Friendly	-0.184	Irritable	-0.103
Distressed	-0.072	Active	-0.276	Agitated	-0.112
Calm	-0.230	Positively occupied	-0.475	Apathetic	-0.122
Friendly	-0.428	Content	-0.713	Uneasy	-0.131
Apathetic	-0.472	Relaxed	-0.752	Frustrated	-0.359
Sociable	-0.556	Calm	-0.774	Content	-0.363

Goat assessment results

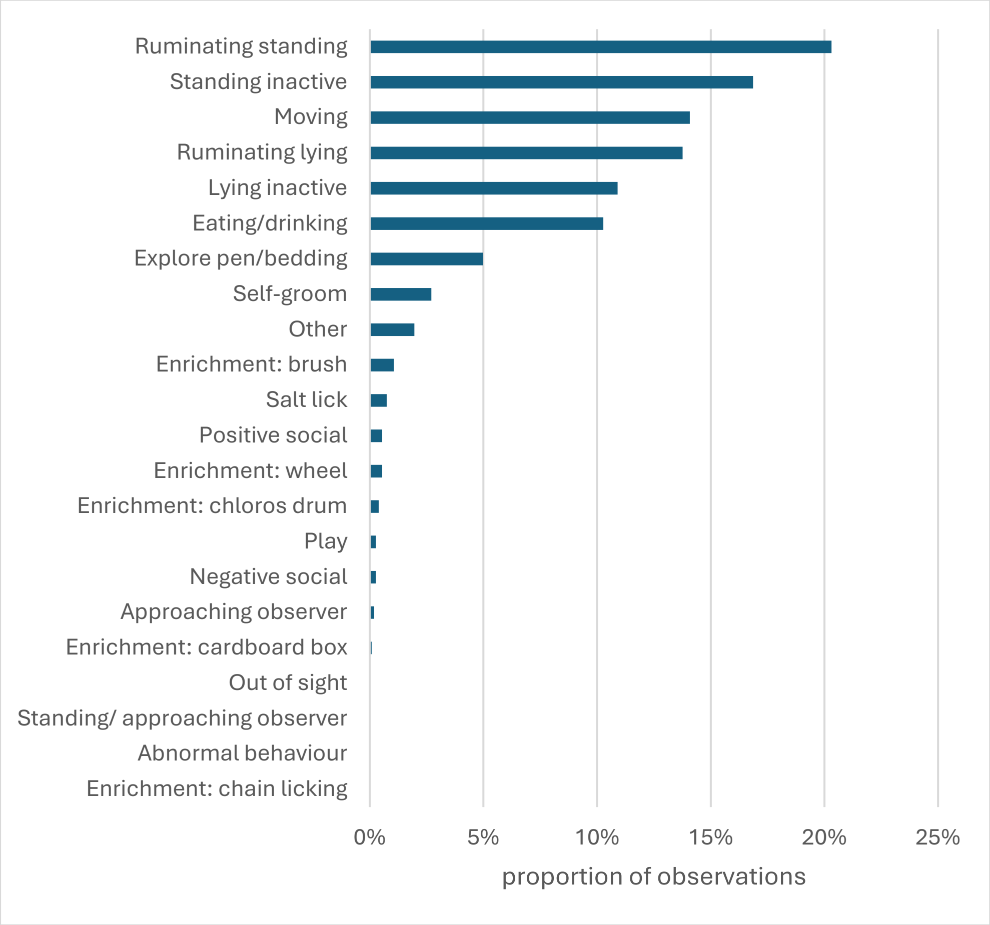

Figure S3: The proportion of observations that goats (n=3) spent performing each behaviour (ruminating standing; standing inactive; moving; ruminating lying; lying inactive; eating/drinking/ explore pen/bedding/ self-groom; enrichment: brush; salt lick; positive social; enrichment: wheel; enrichment: empty disinfectant drum; play; negative social; approaching observer; enrichment: cardboard box; standing/approaching observer; abnormal behaviour; enrichment: chain licking; out of sight; other) during observations (n=28 observation sessions).

QBA results

Principal component analysis (PCA, covariance matrix, no rotation) was conducted in SPSS vs27 on QBA term scores to derive the loadings of each term on the major components.

Total Variance Explained

Component		Initial Eigenvalues[a]			Extraction Sums of Squared Loadings		
		Total	% of Variance	Cumulative %	Total	% of Variance	Cumulative %
Raw	1	3720.673	29.684	29.684	3720.673	29.684	29.684
	2	2291.479	18.282	47.965	2291.479	18.282	47.965
	3	1266.811	10.107	58.072	1266.811	10.107	58.072
	4	1061.906	8.472	66.544	1061.906	8.472	66.544
	5	910.170	7.261	73.805	910.170	7.261	73.805
	6	643.515	5.134	78.939	643.515	5.134	78.939
	7	499.188	3.983	82.922			
	8	392.141	3.129	86.050			
	9	362.152	2.889	88.940			
	10	302.633	2.414	91.354			

11	251.592	2.007	93.361			
12	185.203	1.478	94.839			
13	152.563	1.217	96.056			
14	135.373	1.080	97.136			
15	104.398	0.833	97.969			
16	84.842	0.677	98.646			
17	64.033	0.511	99.157			
18	50.734	0.405	99.561			
19	40.146	0.320	99.882			
20	14.827	0.118	100.000			

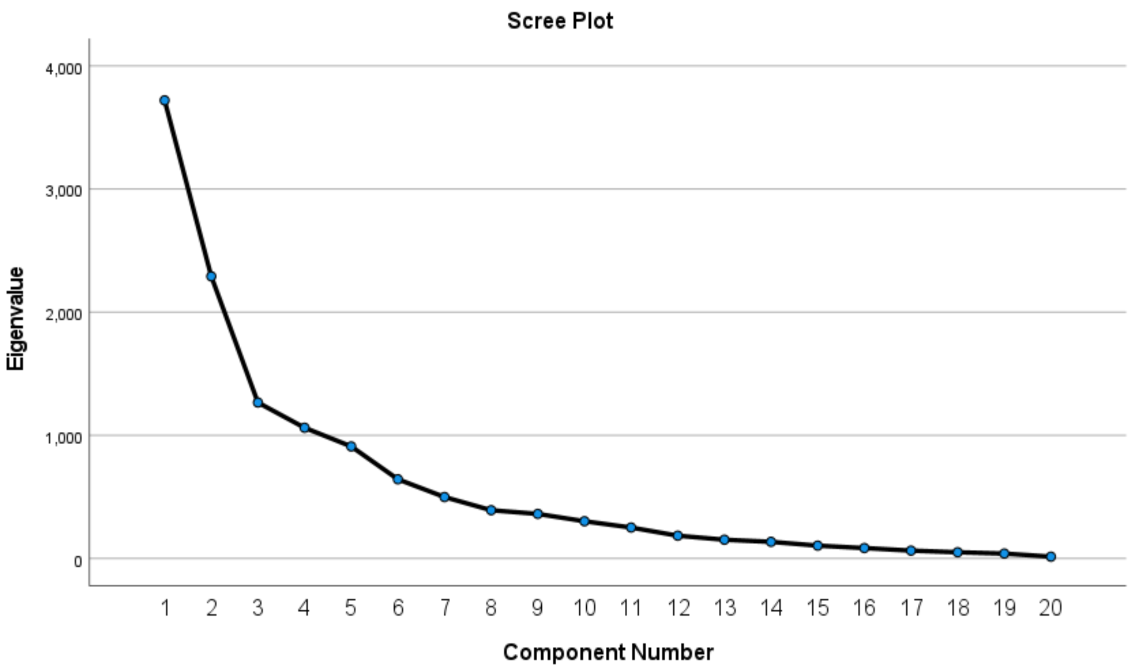

Loadings of individual terms on each component

	Component 1		Component 2		Component 3
Active	0.815	Uneasy	0.668	Friendly	0.774
Playful	0.814	Agitated	0.594	Sociable	0.662
Inquisitive	0.787	Indifferent	0.549	Bored	0.294
Lively	0.752	Bored	0.484	Playful	0.276
Positively occupied	0.649	Fearful	0.451	Fearful	0.242
Irritable	0.604	Frustrated	0.398	Inquisitive	0.200
Agitated	0.519	Irritable	0.349	Active	0.197
Frustrated	0.473	Distressed	0.276	Lively	0.182
Happy	0.395	Apathetic	0.188	Indifferent	0.154
Relaxed	0.329	Playful	0.133	Positively occupied	0.075
Uneasy	0.322	Inquisitive	0.063	Happy	0.044

Bored	0.066	Happy	0.048	Relaxed	0.017
Fearful	-0.010	Lively	-0.050	Distressed	-0.010
Content	-0.021	Sociable	-0.061	Calm	-0.012
Indifferent	-0.027	Friendly	-0.173	Irritable	-0.101
Distressed	-0.091	Active	-0.280	Agitated	-0.111
Calm	-0.187	Positively occupied	-0.457	Apathetic	-0.120
Friendly	-0.416	Content	-0.675	Uneasy	-0.130
Apathetic	-0.475	Relaxed	-0.737	Frustrated	-0.358
Sociable	-0.556	Calm	-0.750	Content	-0.382
